# Interstitial pneumonia and pulmonary hypertension associated with suspected ehrlichiosis in a dog

**DOI:** 10.1186/s13028-016-0228-1

**Published:** 2016-07-07

**Authors:** Marjolein Lisette den Toom, Tetyda Paulina Dobak, Els Marion Broens, Chiara Valtolina

**Affiliations:** 1 Department of Clinical Sciences of Companion Animals, Faculty of Veterinary Medicine, Utrecht University, Yalelaan 108, 3508 TD Utrecht, The Netherlands; 2 Department of Diagnostic Imaging, Faculty of Veterinary Medicine, Utrecht University, Yalelaan 108, 3508 TD Utrecht, The Netherlands; 3 Department of Infectious Diseases and Immunology, Faculty of Veterinary Medicine, Utrecht University, PO Box 80.165, 3508 TD Utrecht, The Netherlands

**Keywords:** Canine, Ehrlichiosis, Pulmonary hypertension, Reversible, Interstitial lung disease

## Abstract

**Background:**

In dogs with canine monocytic ehrlichiosis (CME), respiratory signs are uncommon and clinical and radiographic signs of interstitial pneumonia are poorly described. However, in human monocytic ehrlichiosis, respiratory signs are common and signs of interstitial pneumonia are well known. Pulmonary hypertension (PH) is classified based on the underlying disease and its treatment is aimed at reducing the clinical signs and, if possible, addressing the primary disease process. PH is often irreversible, but can be reversible if it is secondary to a treatable underlying etiology. CME is currently not generally recognized as one of the possible diseases leading to interstitial pneumonia and secondary PH in dogs. Only one case of PH associated with CME has been reported worldwide.

**Case presentation:**

A seven-year-old, male intact, mixed breed dog was presented with 2 weeks history of lethargy and dyspnea. The dog previously lived in the Cape Verdean islands. Physical examination showed signs of right-sided congestive heart failure and poor peripheral perfusion. Thoracic radiography showed moderate right-sided cardiomegaly with dilation of the main pulmonary artery and a mild diffuse interstitial lung pattern with peribronchial cuffing. Echocardiography showed severe pulmonary hypertension with an estimated pressure gradient of 136 mm Hg. On arterial blood gas analysis, severe hypoxemia was found and complete blood count revealed moderate regenerative anemia and severe thrombocytopenia. A severe gamma hyperglobulinemia was also documented. Serology for *Ehrlichia canis* was highly positive. Treatment with oxygen supplementation, a typed packed red blood cell transfusion and medical therapy with doxycycline, pimobendan and sildenafil was initiated and the dog improved clinically. Approximately 2 weeks later, there was complete resolution of all clinical signs and marked improvement of the PH.

**Conclusion:**

This report illustrates that CME might be associated with significant pulmonary disease and should be considered as a possible differential diagnosis in dogs presenting with dyspnea and secondary pulmonary hypertension, especially in dogs that have been in endemic areas. This is important because CME is a treatable disease and its secondary lung and cardiac manifestations may be completely reversible.

## Background

*Ehrlichia canis* is a pleomorphic bacterium that infects circulating monocytes and can cause canine monocytic ehrlichiosis (CME). CME results in variable nonspecific clinical manifestations and clinical signs can be subclinical, acute or chronic. Most dogs present with depression, lethargy, mild weight loss, anorexia, splenomegaly, and lymphadenopathy with or without hemorrhagic tendencies [1, 2]. Respiratory signs are sporadically reported in dogs but are regularly described in human patients infected with human monocytic ehrlichiosis (HME) [3].

Interstitial pneumonia can have an infectious or non-infectious etiology. In dogs, reported infectious agents leading to interstitial pneumonia are *Angiostrongylus vasorum, Leishmania chagasi, Toxoplasma gondii, Pneumocystis carinii, Babesia canis, Leptospira* sp*., Mycoplasma* sp, canine distemper virus and adenovirus [4–12]. In patients with interstitial pneumonia, gas exchange is often impaired due to ventilation-perfusion mismatching, intrapulmonary shunting, and decreased diffusion across the abnormal interstitium with arterial hypoxia as a consequence. In contrast to the systemic vasculature that responds with arterial vasodilation to better perfuse hypoxic tissue, the pulmonary vasculature constricts in response to hypoxia. Besides pulmonary vasoconstriction, hypoxia also causes proliferation of the smooth muscle cells in the arterial wall. Both phenomena lead to a decrease in luminal cross-sectional area and an increase in pulmonary vascular resistance index with pulmonary hypertension (PH) as a consequence.

Pulmonary hypertension is classified based on the underlying disease and its treatment is aimed at improving the clinical signs and addressing the primary disease process [13]. Although PH is often irreversible, PH is reversible in some cases if the underlying etiology is diagnosed and treated accordingly. Reversibility of PH has for instance been demonstrated in dogs after successful treatment for *A. vasorum* [14].

Pulmonary changes consistent with interstitial pneumonia have been reported previously in humans with HME [3] and as an atypical finding in dogs with CME [15–18]. However, CME is generally not recognized as one of the possible diseases leading to interstitial pneumonia and secondary PH in dogs. Only one case of PH associated with *E. canis* infection has been reported worldwide [19]. Consequently, CME might be underdiagnosed as a possible cause of interstitial pneumonia and secondary PH.

This case report describes the clinical, radiographic and echocardiographic presentation of a dog with interstitial pneumonia and severe PH suspected to be associated with *E. canis* infection.

## Case presentation

A seven-year-old, intact male, mixed breed dog weighing 8.1 kg was presented to the Emergency Service of the Department of Clinical Science of Companion Animals of the Faculty of Veterinary Medicine, Utrecht University with a 2 weeks history of lethargy, progressive dyspnea and coughing. The dog previously lived in the Cape Verdean islands for approximately 3 years and returned to the Netherlands 10 months before presentation. In the past 2 years, the dog had showed chronic mild exercise intolerance and had a few episodes of diarrhea that resolved with symptomatic therapy. The dog was up-to date with his vaccinations and anthelminthic treatments.

Physical examination showed generalized weakness and decrease mental state. Cardiovascular examination revealed tachycardia, weak peripheral pulses, pale mucous membranes, prolonged capillary refill time, jugular distensions and venous pulses, and a grade three out of six systolic murmur with the point of maximal intensity over the right cardiac apex. The dog was also severely dyspneic and demonstrated harsh lung sounds on auscultation. The abdomen was distended and positive undulation was detected. These findings were consistent with pulmonary disease, right-sided heart failure and poor peripheral perfusion.

Complete blood count (CBC) showed a moderate microcytic, hypochromic anemia, moderate leukocytosis with a marked left shift and a severe thrombocytopenia. Biochemistry showed severe hyperproteinemia, hyperglobulinemia and a mild hypoalbuminemia. Serum protein electrophoresis showed a polyclonal peak in the gamma globulin region. Arterial blood gas analysis showed a severe hypoxemia with hypocapnia. Urinalysis showed mild hemoglobinuria, glucosuria and proteinuria. Blood samples were submitted for serological and molecular biological testing. Immunofluorescence antibody test (IFAT) for *E. canis* (MegaFLUO Ehrlichia canis^®^, Mega Cor Diagnostik GmbH, Hörbranz, Austria) was positive (IgG titer >2560), but polymerase chain reaction (PCR) amplification for *Ehrlichia* genus (real-time PCR, Light Cycler^®^ 2.0, Roche Diagnostics GmBH, Mannheim, Germany, primers used as described previously [20]) was negative. Serology for *Leishmania* sp. (Dog-DAT^®^, Leishmania specific antibody detection kit, Koninklijk Instituut voor de Tropen, Amsterdam, the Netherlands) and *B.* *canis*. (MegaFLUO Babesis canis^®^, Mega Cor Diagnostik GmbH, Hörbranz, Austria) and antigen snap tests for *A. vasorum* (Angio Detect™ Test, IDEXX Laboratories) and *Dirofilaria immitis* (SNAP^®^ Heartworm RT Test, IDEXX Laboratories) were also negative. Laboratory results are summarized in Table 1.

**Table 1:** Summary of haematological, biochemical, serological and urine and blood gas analysis results

Parameter	Day 1	Day 7	Day 17	Day 50	Reference interval
Hematocrit (L/L)	*0.20*	*0.38*	*0.40*	*0.34*	0.42–0.61
MCV (fl)	*57.7*		*59.9*	*59.9*	63.5–72.9
MCHC (mmol/L)	21.7		21.8	22.2	20.5–22.4
MCH (fmol)	*1.25*		*1.31*	*1.33*	1.37–1.57
Total WBC (×10^9^/L)	*23.1*		6.6	6.7	4.5–14.6
Segmented neutrophils (×10^9^/L)	*17.3*		2.6	4.4	2.9–11.0
Band neutrophils (×10^9^/L)	*3.2*		0.0	0.0	0.0–0.3
Lymphocytes (×10^9^/L)	2.1		3.0	1.8	0.8–4.7
Monocytes (×10^9^/L)	0.5		0.4	0.3	0.0–0.9
Eosinophils (×10^9^/L)	0.0		0.5	0.2	0.0–1.6
Platelets (×10^9^/L)	*15*		251	166	144–603
Urea (mmol/L)	4.1		7.0		3.0–12.5
Creatinine (µmol/L)	33		74	77	50–129
Sodium (mmol/L)	145	145	141		141–150
Potassium (mmol/L)	4.1	4.7	3.7		3.6–5.6
Total protein (g/L)	*91*		*119*	*89*	55–72
Albumin (g/L)	*16*		27	26	26–37
Gamma-globulins (g/L)	*57*		*68*	*40*	3–9
UPC	*0.8*			0.3	<0.5
PH	7.45	*7.49*			7.35–7.45
PaO_2_ (mm Hg)	*48*	*55*			85–103.3
PaCo_2_ (mm Hg)	*19.8*	32			32–43
BE	*−9*	1.4			−2 to +2
Lactate (mmol/L)	*2.6*	1.3			<2.5
IgG titer *Ehrlichia Canis*	*>1:2560*		*1:2560*		<1:40

Arterial blood gas analysis was performed with an inspired concentration of oxygen of 21 %

Values in italics are outside the reference interval

*MCV* mean corpuscular volume, *MCHC* mean corpuscular hemoglobin concentration, *MCH* mean corpuscular hemoglobin, *WBC* white blood cell count, *UPC* urinary protein to creatinine ratio, *PaO*_*2*_ partial arterial oxygen pressure, *PaCO*_*2*_ partial arterial carbon dioxide pressure, *BE* base excess

**Fig. 1:**
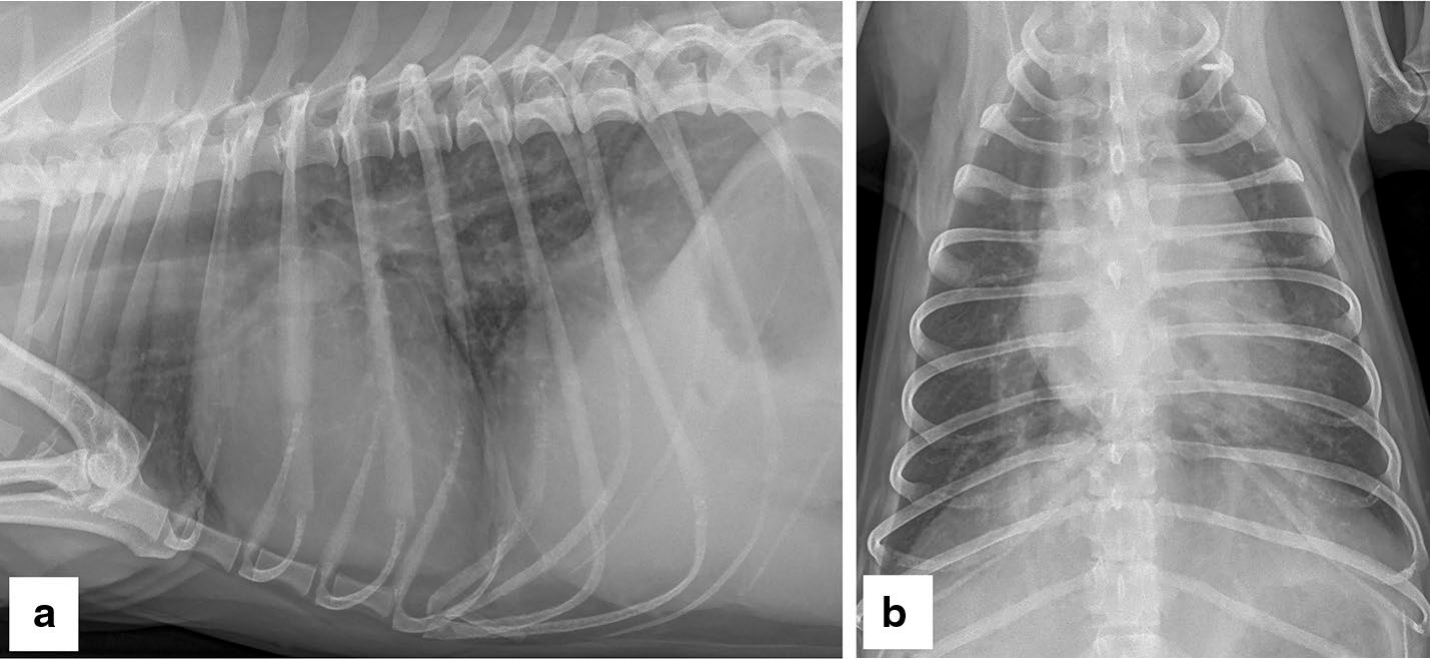
Right lateral (**a**) and dorsoventral (**b**) thoracic radiographs at presentation. Radiographs demonstrating right-sided enlargement of the cardiac silhouette (vertebral heart score: 11.0, reference interval <9.7 ± 0.5), mild dilation of the pulmonary arteries and a mild increase in lung opacity with a diffuse interstitial lung pattern and peribronchial cuffing

On thoracic radiographs, a mild diffuse increase in pulmonary opacity with an interstitial lung pattern and mild peribronchial cuffing was seen, which was most accentuated in the caudodorsal lung lobes. Thin pleural fissure lines were noted between all lung lobes. The cardiac silhouette showed signs of right-sided cardiomegaly (vertebral heart score (VHS): 11.0, reference interval <9.7 ± 0.5) and a main pulmonary artery knuckle was present on the dorsoventral view (Fig. 1).

**Fig. 2:**
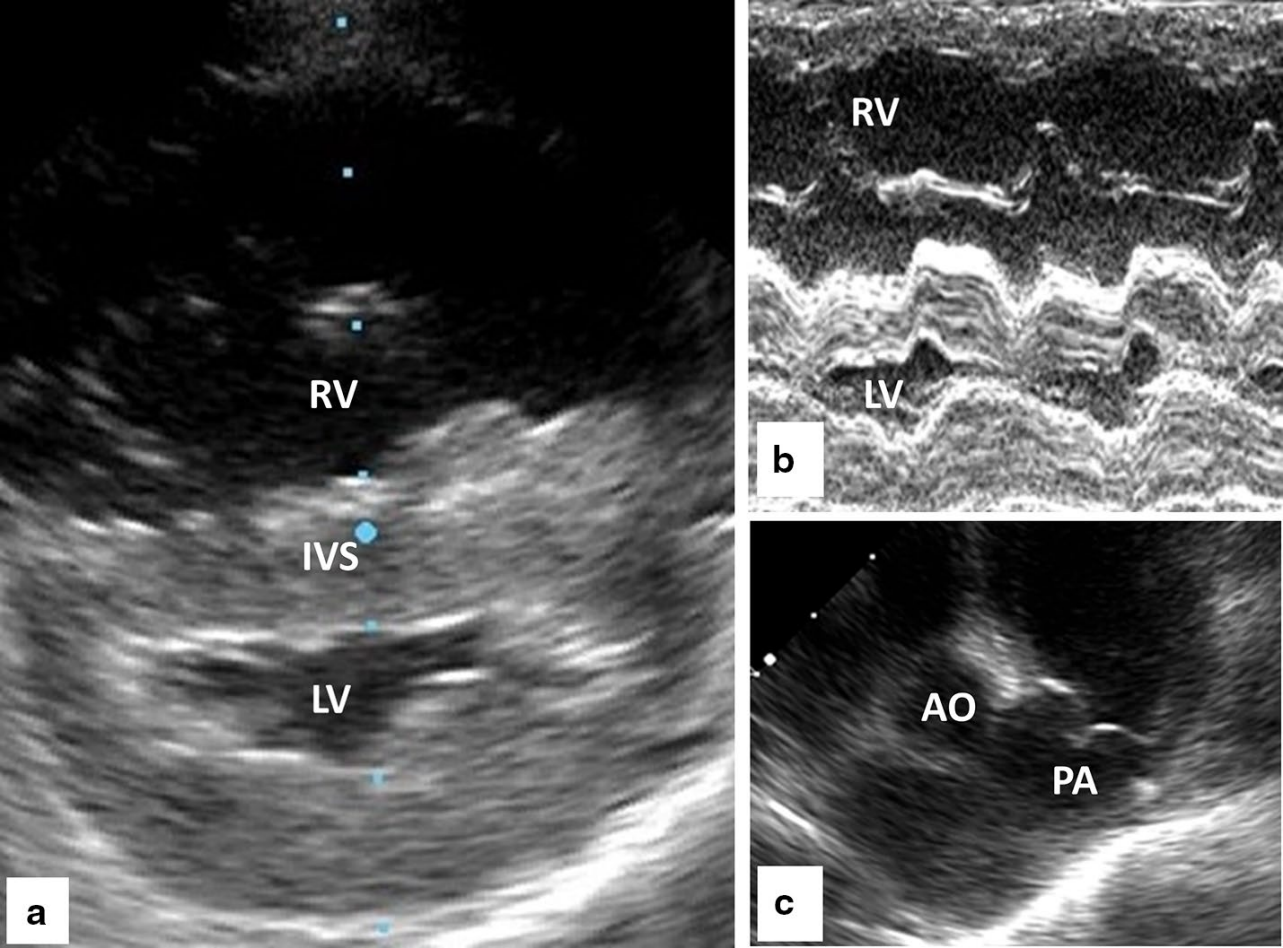
Echocardiographic images at presentation from right parasternal short axis view. **a** Two-dimensional view demonstrating severe right ventricular (RV) dilation, flattening of the interventricular septum (IVS) and a hypovolemic left ventricle (LV). **b** M-mode view demonstrating severe right ventricular dilation, a hypovolemic left ventricle and paradoxical motion of the interventricular septum. **c** Two-dimensional view of pulmonary artery (PA) and aorta (AO). The PA is uniformly dilated and is wider than the Aorta with a PA/AO ratio of 1.3 (reference interval: 0.8–1.15)

**Fig. 3:**
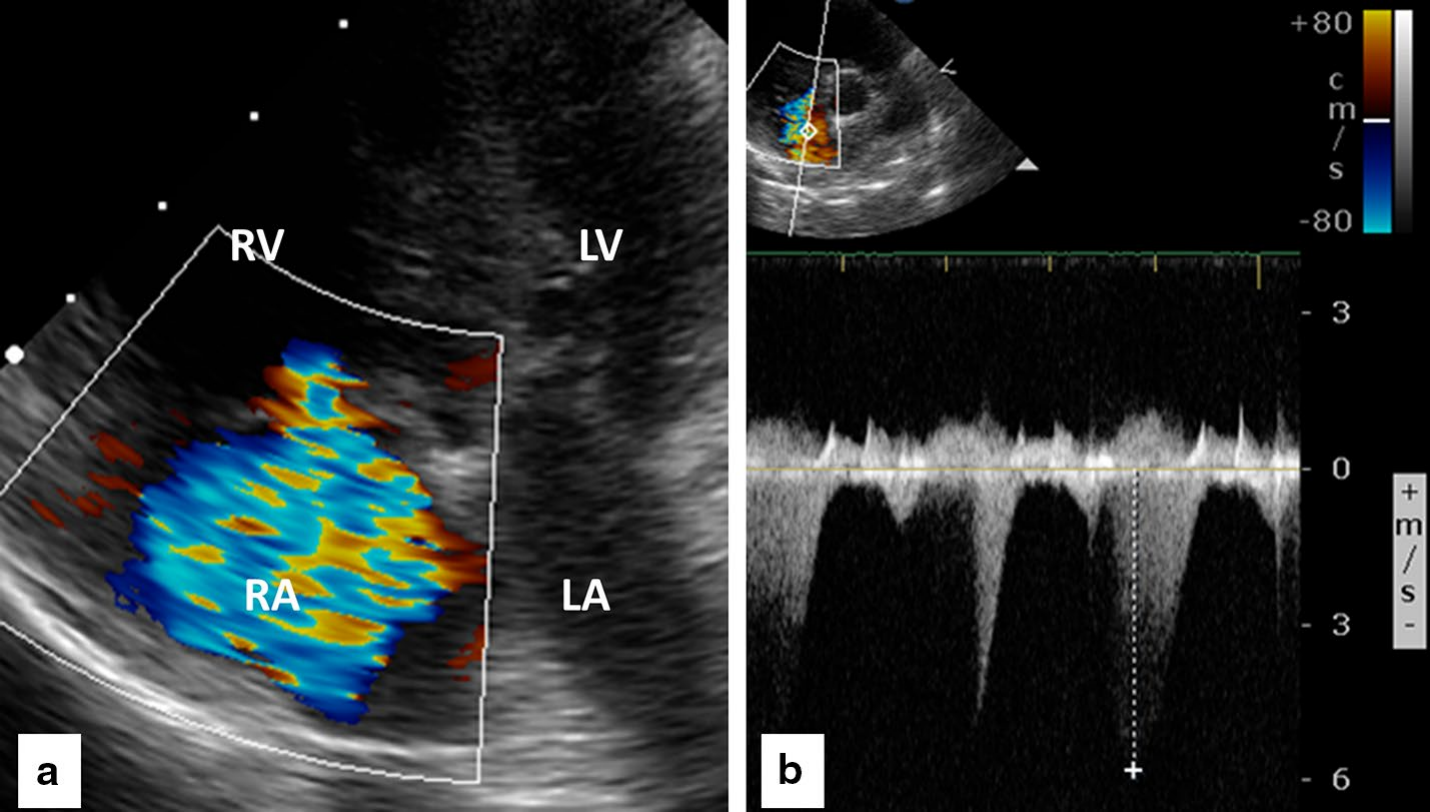
Echocardiographic images at presentation from left apical 4-chamber view. **a** color Doppler map of severe tricuspid regurgitation. *RA* right atrium, *RV* right ventricle, *LA* left atrium, *LV* left ventricle. **b** spectral Doppler trace of tricuspid regurgitation. Tricuspid systolic velocity of 5.8 m/s, indicating a peak tricuspid pressure gradient of approximately 136 mm Hg, graded as severe pulmonary hypertension (reference <40 mm Hg, severe >75 mm Hg

Echocardiography showed dilation of the right ventricle and the pulmonary artery, septal flattening and a severe tricuspid regurgitation. The left ventricle was severely under filled (Fig. 2). Congenital defects and left heart disease were excluded. The maximal tricuspid systolic velocity was 5.83 m/s, indicating a peak tricuspid gradient of approximately 136 mm Hg which is graded as severe PH (reference <40 mm Hg, severe >75 mm Hg) [13], (Fig. 3).

To address the severe hypoxemia and PH the dog was placed in an oxygen cage with an inspired concentration of oxygen between 40 and 50 %. The clinical signs of the dog did not improve markedly with the extra oxygen supplementation. Because anemia could have contributed to the cardiovascular signs and the poor tissue oxygenation, a typed packed red blood cell transfusion was administered. Based on the travel history, the abnormalities within the CBC and biochemical analysis and the positive serology for *E. canis*, an infection with CME was suspected. Treatment with doxycycline (5 mg/kg, orally twice daily) (Doxoral^®^, AST Farma, Oudewater, the Netherlands), pimobendan (0.3 mg/kg, orally twice daily) (Cardisure^®^ Flavour, Eurovet Animal Health BV, Bladel, the Netherlands) and sildenafil (1.5 mg/kg, orally twice daily) (Viagra^®^, Pfizer, New York, USA) was initiated.

The hematocrit increased from 20 to 36 % after the blood transfusion and the dog’s clinical condition improved remarkably. However, the dog remained moderately dyspneic and severely hypoxic (PaO_2_: 46.5 mm Hg, reference interval: 85.0–103.3 mm Hg). The dyspnea gradually improved and the dog seemed comfortable outside the oxygen cage after 6 days of treatment, although the improvement of the hypoxemia was only minimal (PaO_2_: 55 mm Hg, reference interval: 85–103.3 mm Hg, Table 1). Because of financial limitations of the owner, the dog was discharged with the above-mentioned therapies at that time.

**Fig. 4:**
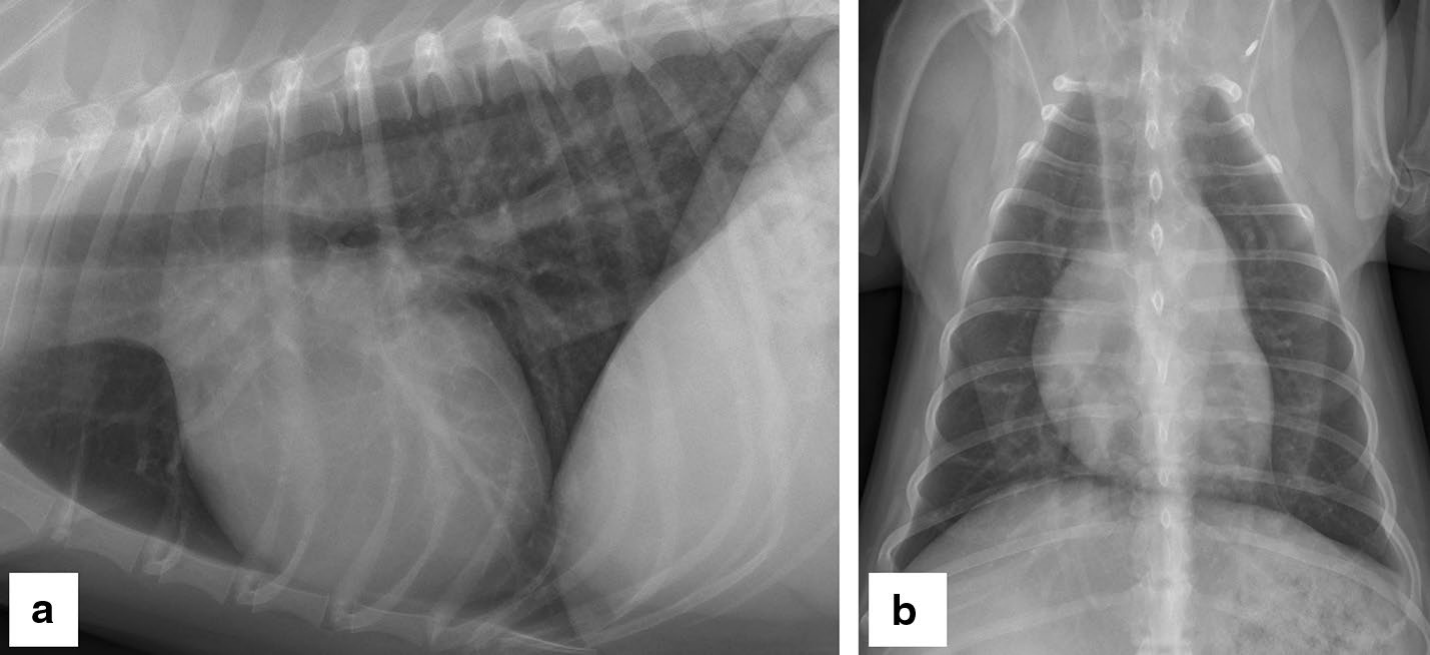
Right lateral (**a**) and dorsoventral (**b**) thoracic radiographs 2 weeks after discharge. Radiographs demonstrating resolution of cardiomegaly (vertebral heart score: 10.2, reference interval <9.7 ± 0.5) and reduction of the dilation of pulmonary arteries and the diffuse interstitial pattern

**Fig. 5:**
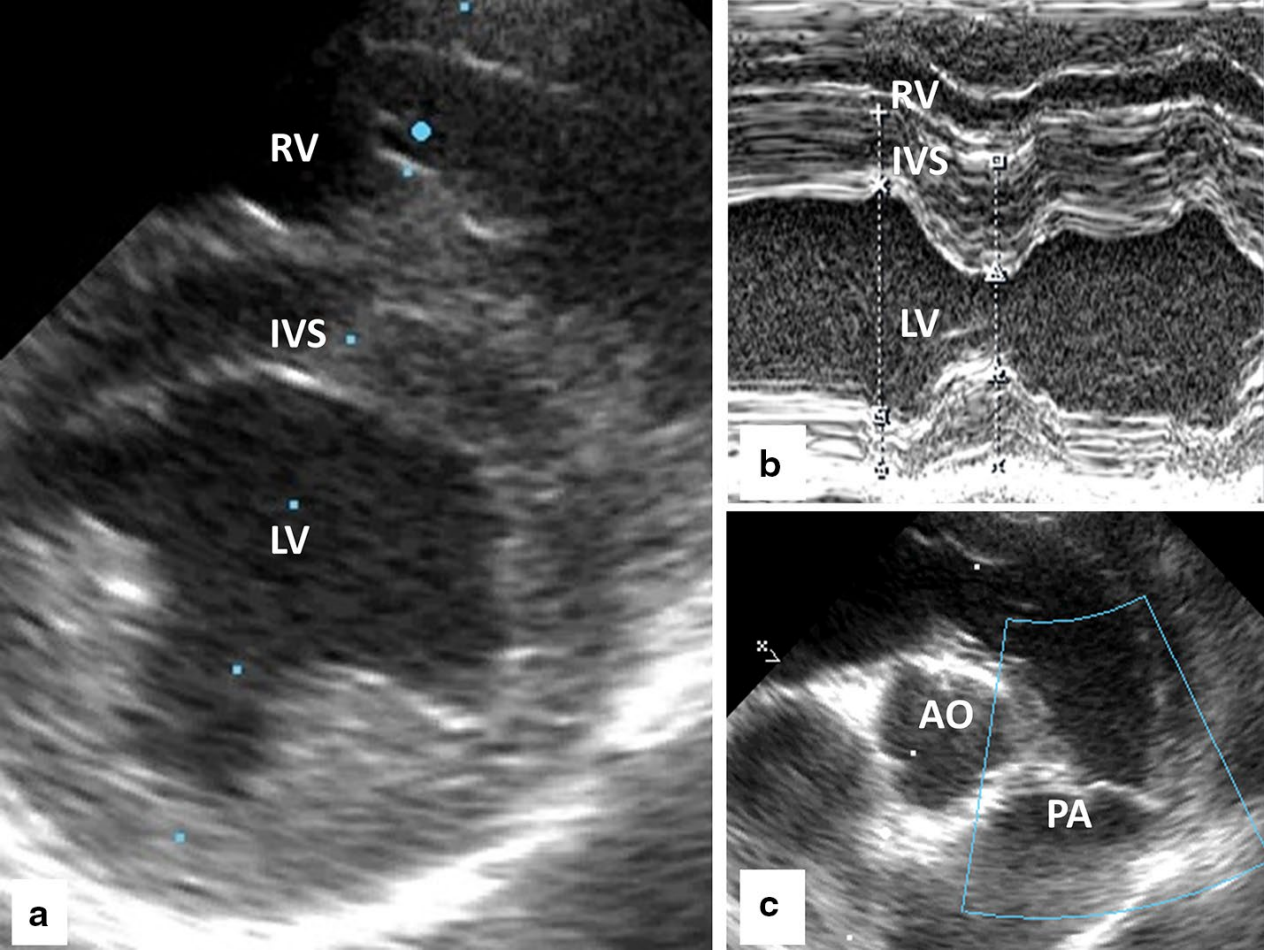
Echocardiographic images obtained 2 weeks after treatment from right parasternal short axis view. **a**, **b**: Two-dimensional (**a**) and M-mode (**b**) view demonstrating normalization of cardiac dimensions and function. **c** Two-dimensional view of pulmonary artery (PA) and aorta (AO). The PA is uniformly minimally dilated with a PA/Ao ratio of 1.2 (reference interval: 0.8–1.15)

Seventeen days after initiation of treatment, the dog was admitted via the cardiology polyclinics of the same university and re-examined. At that time, there was complete resolution of all clinical signs and physical examination was completely unremarkable. CBC showed normal platelet counts and leukogram, with only a very mild microcytic hypochromic anemia. Biochemical analysis again showed severe hyperproteinemia and hyperglobulinemia. The albumin concentration normalized. The IgG titer for *E. canis* (IFAT) was again very high (IgG titer 2560) (Table 1). Thoracic radiography showed marked improvement with resolution of cardiomegaly (VHS: 10.2, reference interval 9.7 ± 0.5) and only a very mild interstitial pattern of the caudodorsal lung lobes and a very mild dilation of the pulmonary artery (Fig. 4) as remaining abnormalities. Echocardiography also showed a remarkable improvement. Tricuspid regurgitation was no longer present and only a very mild uniform dilation of the pulmonary artery was still present (Fig. 5). Therapy with pimobendan and sildenafil were discontinued and the treatment with doxycycline was continued for another 5 weeks.

The dog was reevaluated 4 weeks later and was clinically doing very well. Nevertheless, the dog again developed mild anemia and the hyperproteinemia persisted. Proteinuria had resolved at that time (Table 1). Doxycycline therapy was continued for another 4 weeks and another re-examination was advised. Unfortunately, the dog was lost to follow–up at that time.

## Conclusion

Interstitial pneumonia with secondary PH was suspected in this dog based on the presentation on thoracic radiography and the severe hypoxemia on arterial blood gas, but the etiology was initially unclear. However, CME was suspected based on the travel history of the dog, the results of CBC, biochemical analysis and a positive serology result for *E. canis*. Exposure to *E. canis* was demonstrated with the IFAT for anti-*E. canis* IgG antibodies. IgG titers >40 are considered positive for *E. canis* exposure [21]. In this case, the IgG titer was high (>2560), which strengthened the suspicion of an active infection [22].

However, definite active infection at the time of presentation could not be proven, because PCR amplification for *Ehrlichia* genus was negative and anti-*Ehrlichial* IgG antibodies persist for several months to years after elimination of the parasite [23]. A negative result from a PCR test can occur when organisms in circulation are below the level of detection, as may happen when infections are chronic. It has been demonstrated that dogs can be PCR negative on blood samples, but PCR positive on splenic aspirates [21]. It is hypothesized that the *E. canis* are sequestered in splenic macrophages to avoid immune elimination. Unfortunately, in the present case, the owners declined fine needle aspirates of the spleen due to the potential risks and stress involved, e.g. internal bleeding, aggravation of the dyspnea. Paired serology samples can also provide useful information about antibody kinetics, which may point to current status of infection. A four-fold increase in IgG antibodies over time is suggested to be evidence for an active infection [24]. Antibody titers will decrease gradually after appropriate treatment, but may persist for months to years even after full clinical recovery [23, 25]. In this case, the *Ehrlichia* IgG titer was still very high 17 days after initiation of doxycycline therapy, probably due to the short time between start of the treatment and retesting; IFAT was unfortunately not repeated on day 50.

Although doxycycline therapy generally results in fast clinical improvement and improvement of most laboratory abnormalities, persistence of hyperglobulinemia is generally observed for a longer period. Most studies have shown normalization of serum protein electrophoresis results after 3–9 months of therapy [1, 15]. This explains the persistence of hyperglobulinemia at the re-examinations of the dog at days 17 and 50.

In human medicine, respiratory signs are commonly described as a consequence of HME infection [3] and acute respiratory distress syndrome (ARDS) has also been reported as a severe, although uncommon, finding in HME [26–28]. Both CME and HME animal models also revealed prominent mononuclear cellular infiltration in the interalveolar septa, endothelial damage and vasculitis in the lungs [16, 29]. This could explain the pulmonary changes found in this dog and the clinical and radiographic improvement after treatment with doxycycline. However, recently, acute resolution of patchy pulmonary alveolar infiltrates has been described after sildenafil therapy in dogs with idiopathic PH and PH secondary to idiopathic lung fibrosis [30]. Therefore, sildenafil therapy might also have contributed to the improvement of the radiographic changes in this case. Another explanation for the radiographic abnormalities and the improvement on doxycycline therapy that cannot be excluded is that the dog suffered from a bacterial pneumonia.

Furthermore, an important possible factor that might have contributed to the hypoxemia and PH in this dog is pulmonary thromboembolism (PTE) [31]. PTE can occur as complicating sequelae in patients with PH or can be the primary cause of PH [32]. In people, it is increasingly recognized that patients with pulmonary arterial hypertension have dysregulated coagulation and antithrombotic homeostasis, which may contribute to a prothrombotic state [33]. Unfortunately, we did not perform diagnostic investigations such as D-dimer concentration [34], computed tomography pulmonary angiography [35] or thromboelastography (TEG) [36, 37] to investigate if the dog suffered from PTE or a prothrombotic state. However, to the author’s knowledge, PTE has never been associated with CME in veterinary literature. Only two cases of aortic and portal vein thrombosis have been described in dogs with CME [38, 39].

Treatment of PH is aimed at eliminating or improving the underlying disease process. If the PH is not controlled by primary disease therapy or if the PH is idiopathic, treatment with pulmonary arterial dilators may be implemented. In veterinary medicine currently, only phosphodiesterase inhibitors are used. Sildenafil is a highly selective phosphodiesterase five inhibitor that has been used in veterinary medicine with encouraging results [30, 40]. Pimobendan, a calcium-sensitizing agent with phosphodiesterase three inhibiting actions has also been used, especially when left heart disease is a contributing cause [41]. In the present case, a dual therapy with pulmonary arterial dilators was initiated, because we hoped this would ameliorate the very severe PH and clinical signs faster than a monotherapy with sildenafil. Although recovery has been described in a similar case with just a monotherapy with doxycycline [19], we believe that the symptomatic support with pulmonary vasodilators was justified in this dog. Mild to moderate improvement of tricuspid regurgitation gradient by sildenafil therapy has been described in some dogs with PH [30], but not in others [42]. Consequently, we cannot rule out that the improvement of the echocardiographic changes could also be partially explained by the use of the vasodilating drugs. Ideally, another echocardiogram should have been performed after discontinuation of the vasodilator therapy. However, the improvement was so dramatic, that we do not believe that this could be solely explained by the vasodilator therapy.

This case report illustrates that CME might be associated with significant pulmonary disease and that it should be considered as a possible differential diagnosis in dogs presenting with dyspnea and secondary pulmonary hypertension, especially in dogs that have been in endemic areas. This is important because CME is a treatable disease and its lung and cardiac manifestations may be completely reversible.
